# The relevance of combined testing of cerebrospinal fluid glial fibrillary acidic protein and ubiquitin C-terminal hydrolase L1 in multiple sclerosis and peripheral neuropathy

**DOI:** 10.1007/s10072-024-07790-4

**Published:** 2024-11-20

**Authors:** Peter Csecsei, Peter Acs, Marianna Gottschal, Piroska Imre, Egon Miklos, Diana Simon, Szabina Erdo-Bonyar, Timea Berki, Laszlo Zavori, Reka Varnai

**Affiliations:** 1 Department of Neurosurgery, Medical School, University of Pecs, Pecs, Hungary; 2 Department of Neurology, Medical School, University of Pecs, Pecs, Hungary; 3 Department of Neurology, Kanizsai Dorottya Hospital, Nagykanizsa, Hungary; 4 Department of Neurology, Veszprém County Csolnoky Ferenc Hospital, Veszprém, Hungary; 5 Department of Neurology, Vas County Markusovszky University Teaching Hospital, Szombathely, Hungary; 6 Department of Immunology and Biotechnology, Medical School, University of Pecs, Szigeti str. 12, Pecs, 7624 Hungary; 7 Emergency Department, Saudi German Hospital, Dubai, United Arab Emirates; 8 Department of Primary Health Care, Medical School University of Pecs, Pecs, Hungary

**Keywords:** Glial fibrillary acid protein, Ubiquitin C-terminal hydrolase, Multiple sclerosis, Peripheral neuropathy

## Abstract

**Introduction:**

This study investigates the significance of glial fibrillary acidic protein (GFAP) and ubiquitin C-terminal hydrolase L1 (UCHL-1) in cerebrospinal fluid (CSF) of patients with multiple sclerosis (MS) and peripheral neuropathy (PN).

**Methods:**

We included 41 MS patients, 35 PN patients, and 36 controls across 5 sites. MS patient data included lesion counts, disease activity, albumin quotient, and Expanded Disability Status Scale (EDSS) scores. PN patients included those with acute and chronic inflammatory demyelinating polyneuropathy and sensorimotor neuropathy based on nerve conduction studies. CSF concentrations of GFAP and UCHL-1 were measured using the MILLIPLEX Map Human Neuroscience Magnetic Bead Panel 1.

**Results:**

Both GFAP and UCHL-1 levels were significantly higher in the two patient groups compared to controls. In the MS group, GFAP showed a strong correlation with disease duration, EDSS score, non-enhancing lesions, and the CSF/blood albumin quotient. UCHL-1 levels were significantly higher in patients with active disease (gadolinium-enhancing lesions). The combination of UCHL-1 and GFAP improved diagnostic accuracy (AUC 0.895, 95% CI 0.780-1.000) compared to the independent measurement of either marker for indicating Gd-negative lesions. In the PN group, CSF GFAP levels were significantly lower in patients with purely demyelinating neuropathy compared to those with axonal or mixed neuropathy.

**Conclusion:**

GFAP serves as a sensitive marker for axonal damage in PN, while UCHL-1 closely correlates with disease activity in MS patients.

## Introduction

Multiple sclerosis, a neuroinflammatory disease of the central nervous system that leads to demyelination and neuronal injury, is among the most frequent causes of non-traumatic disability in young adults aged 18 to 40 years and it places a significant burden on the healthcare system [1, 2]. Despite extensive research on various biomarkers [3], a highly sensitive marker that accurately indicates disease activity, relapse, or response to therapy has not yet been identified. Astrocytes are essential for the normal functioning of synapses and play a role in maintaining axonal metabolism by regulating ion homeostasis [4]. Glial fibrillary acidic protein (GFAP) is a type III intermediate filament (IF) protein exclusively located in astrocytes within the central nervous system, non-myelinating Schwann cells in the peripheral nervous system, and enteric glial cells [5].

GFAP is a useful and sensitive marker for various conditions involving nervous system damage, such as TBI [6], intracerebral hemorrhage [7], and common neurodegenerative diseases, including Alzheimer’s disease, prion diseases, frontotemporal lobar degeneration, and Parkinson’s disease [8, 9]. GFAP is linked to greater disabilities and more frequent relapses in MS patients [10]. The level of CSF-GFAP correlates with MS and its subtypes, reflecting the varying degrees of astrocyte damage. Furthermore, progressive MS is more strongly associated with increased cerebrospinal fluid GFAP levels compared to relapsing-remitting MS, suggesting that GFAP could serve as a useful marker for disease progression [11]. UCHL1 is a stable, neuron-specific protein whose levels rise in serum and cerebrospinal fluid (CSF) after traumatic brain injury, correlating with the severity of the injury and long-term outcomes [6, 12]. As a highly abundant deubiquitinating enzyme in the brain, UCHL1 is crucial for nervous system function [13]. It plays a role in repairing damaged axons and neurons and is also involved in immune responses [14]. The concentration of UCHL1 in the blood may indicate the size and location of central nervous system (CNS) damage [15]. Gorska et al. suggest that UCHL1 could be a highly sensitive biomarker for differentiating MS patients from healthy individuals [16]. Peripheral neuropathy’s pathophysiology arises from damage to small- or large-diameter nerve fibers. This damage can affect the cell body, axon, myelin sheath, or a combination of these structures, resulting in symptoms like numbness, tingling, pain, and weakness [17]. Although peripheral neuropathies have various etiologies and triggering factors, most lead to axonal damage and dysfunction at varying rates, ultimately resulting in peripheral nerve damage [18]. Accurately determining the extent and degree of axonal damage remains challenging even today. A more precise determination of the extent of axonal damage could influence treatment strategies, rehabilitation, and prognosis. GFAP levels have been found to be elevated in critical illness polyneuropathy following COVID-19, and these levels correlate with nerve amplitudes. Consequently, GFAP could play a role in diagnosing and predicting axonal nerve pathology [19]. UCHL1 is highly expressed in neurons, particularly sensory neurons. Loss of UCHL1 leads to axonal degeneration in leg sensory neurons and results in diabetic sensory neuropathy [20]. Serum GFAP showed a significant correlation with summated sensory nerve action potential amplitudes and disease severity in chronic neuropathies [21]. These findings suggest using serum GFAP as a marker for axonal damage and disease severity in chronic neuropathies.

GFAP and UCHL-1 together have very high sensitivity for intracranial traumatic injury [6], and a diagnostic kit containing these two molecules is already commercially available [22]. This study confirmed the high sensitivity of GFAP and UCHL-1 for CT abnormalities in mTBI patients using this combined test [12]. Given the combined sensitivity of these two molecules for traumatic CNS injuries and other neurological diseases, our study aims to evaluate the combined predictive value of these two molecules in patients with multiple sclerosis or peripheral neuropathy.

## Methods

### Study design and patients

In this prospective study, CSF samples were collected from 41 MS and 35 PN patients attending four County Hospitals (Veszprem, Nagykanizsa, Szombathely, Baja) in Hungary and the Department of Neurology at the University of Pecs between 2023 and 2024 as a part of routine diagnostic workups. For the MS patients, the 2017 revision of the McDonald criteria were applied [23]. The clinical severity was measured by assessing the EDSS [24] for MS patients and the GBS disability scale [25] and the Rasch-built Overall Disability Scale [26] for PN patients. A standard MRI protocol for MS with IV gadolinium (Gd) as contrast was used in all centers. The presence of contrast-enhancing T1-weighted lesions was evaluated using MRI data and radiologic reports. We included only MRIs performed 4 weeks before or after lumbar puncture to investigate the influence of disease activity on MRI. The disease activity was defined as a Gd- enhancing lesion on MRI [27]. MS phenotype patients consisted of relapsing-remitting multiple sclerosis (RRMS), progressive multiple sclerosis (PMS, i.e. primary or inactive secondary MS) and clinically isolated syndrome (CIS). Clinical data collected during the examination were as follows: For MS patients, the demographic data (age, gender), the number of Gd-enhancing and non-enhancing MRI lesions, the time elapsed between the first symptoms and the CSF sampling, and the CSF/blood albumin quotient (increased CSF/serum albumin quotient as an indication for blood-brain barrier dysfunction). For PN patients, we recorded the admission CSF protein, CRP, HgBA1C, glucose, creatinine values, and the results of electrophysiological examinations (demyelination, axonal damage, mixed lesions). Additionally, we recorded the time elapsed between the onset of symptoms and the CSF sampling.

Among PN patients, we established the following subgroups [28]: (A) Acute inflammatory demyelinating polyneuropathy (AIDP) patients with peripheral demyelinating neuropathy, demyelination fulfilling clinical, laboratory and electrophysiological criteria for Guillain-Barré syndrome of AIDP type [29], (B) CIDP [30], (C) SMI (axonal sensori-motor “inflammatory” PN with increased CSF protein level (normal range 15–40 mg%) and without electrophysiological signs of demyelination, (D) sensoro-motor “non-inflammatory” PN (CSF protein level within normal range). The nerve conduction studies were performed at all examination sites in accordance with current guidelines [31, 32] by neurologists experienced and licensed to conduct electrophysiological examinations.

The control group consisted of thirty six patients who presented with subjective neurological symptoms but whose diagnostic workup did not reveal an underlying organic disease. Institutional review board approval was obtained previously (BM/5804-1/2024, BM/5804-3/2024), and written informed consent was obtained from each patient or their legal representative.

## CSF examination and assay

The liquor concentration of GFAP and UCHL1 were determined using MILLIPLEX Map Human Neuroscience Magnetic Bead Panel 1 (HNS1MAG-95 K, Merck KGaA, Darmstadt, Germany) according to the manufacturer’s recommendation. Briefly, liquor samples, standards and controls with equal volumes of assay buffer and fluorescent-coded magnetic bead mixture coated with capture antibodies specific for GFAP and UCHL1 were placed in the corresponding wells of the 96-well plate. After overnight incubation, detection of bound analytes was performed with biotinylated detection antibody and Streptavidin-phycoerythrin conjugate. The assay was run with Luminex MAGPIX instrument (Luminex Corporation, Austin, TX, USA). Data were analyzed with Belysa Immunoassay Curve Fitting Software (Merck KGaA, Darmstadt, Germany).

### Statistical analysis

We utilized nonparametric tests, conducting all analyses with SPSS^®^ Statistics version 25 (IBM Corporation, Armonk, NY, USA) and GraphPad Prism 9 software (GraphPad Software, San Diego, USA). The data are presented as medians or means ± SD. All P-values are two-tailed, with values below 0.05 indicating statistical significance. Baseline characteristics between groups were compared using the Kruskal-Wallis test or chi-square test as appropriate. Differences in serum GFAP or UCHL-1 levels between groups were assessed using the Kruskal-Wallis and Dunn tests. Spearman coefficients and partial correlation were calculated to determine correlations. The accuracy of GFAP and UCHL-1 in predicting number of gadolinium negative lesions, EDSS score and disease duration were evaluated by receiver operating characteristic curves, and the data are presented as the area under the curve (AUC).

## Results

### Baseline characteristics

We included a total of 76 patients and 36 controls in the study. Among the patients, 41 (54%) had multiple sclerosis, 35 (46%) had peripheral neuropathy. The mean age of the entire sample was 48 years (SD ± 14), with 42% of the participants being female. Patients with PN had a significantly higher mean age compared to patients with MS (55 ± 14 and 56 ± 11 vs. 42 ± 11, *p* < 0.001). The age of the control group did not significantly differ from the average age of the patient group (49 ± 10 years vs. 48 ± 14, *p* = 0.975). For the MS group the median EDSS was 1.25 (IQR:1-2.25) and more than half of patients were female (51.2%). In the MS group, 70% of the patients (*n* = 28) belonged to the RRMS group, 18% (*n* = 7) to the PPMS group, and 12% (*n* = 5) to the CIS group. The median number of months from first symptoms until the CSF sample was collected was 12 (4–48). In the neuropathic group, based on the distribution of symptoms, 26% of the patients had symmetric symptoms, 9% had upper limb symptoms, 34% had lower limb symptoms, and 31% had symptoms affecting all four limbs. Table 1. summarizes the baseline characteristics of included patients and controls.

**Table 1:** ^a^ The number of months from first symptoms until the CSF sample is collected. MS: multiple sclerosis; PN: peripheral neuropathy; SD: standard deviation; IQR: interquartile range; EDSS: expanded disability status scale; Gd: gadolínium; MRI: magnetic resonance imaging; CSF: cerebospinal fluid; GBS: Guillain-Barré syndrome; CIDP: chronic inflammatory demyelinating polyradiculoneuropathy; AIDP: acute inflammatory demyelinating polyneuropathy; CRP: C-reactive protein; GFAP: glial fibrillary acid protein; UCHL-1: ubiquitin C-terminal hydrolase

	MS (*n* = 41)	PN (*n* = 35)	Control (*n* = 36)
age (mean, SD)	42 ± 11	55 ± 14	49 ± 10
female (n, %)	21 (51.2)	12 (33.3)	21 (58)
disease duration ^a,^ months, median (IQR)	12 (4–48)	5 (2–12)	N/A
EDSS, median (IQR)	1,25 (1–2,25)	N/A	N/A
disease activity - Gd-enhancing lesions in MRI (n, %)	17 (42,5)	N/A	N/A
Non-enhancing lesions in MRI (median, IQR)	12 (6–19)	N/A	N/A
CSF/blood albumin quotient (median, IQR)	6 (4.2–8.2)	7 (5.5–11.6)	N/A
GBS disability scale, (median, IQR)	N/A	3 (2–3)	N/A
Rasch-built Overall Disability Scale, (median, IQR)	N/A	28 (21–36)	N/A
Non-inflammatory PN (n,%)	N/A	5 (14.3)	N/A
Axonal PN (n, %)	N/A	12 (34.3)	N/A
CIDP (n, %)	N/A	11 (31.4)	N/A
AIDP (n, %)	N/A	7 (20)	N/A
Pure demyelination on the electrophysiological studies, (n, %)	N/A	9 (26)	N/A
CSF protein, g/l, median (IQR)	N/A	493 (386–717)	N/A
CRP, mg/L, median (IQR)	N/A	2.6 (2–6)	N/A
HgBA1C, %, median (IQR)	N/A	5.7 (5–6)	N/A
Glucose, mmol/L, median (IQR)	N/A	6.2 (5–7)	N/A
Creatinine, µmol/L, median (IQR)	N/A	76 (60–93)	N/A
GFAP (pg/mL), median (IQR)	282 (202–432)	386 (167–624)	71 (57–115)
UCHL-1 (pg/mL), median (IQR)	431 (229–609)	482 (216–677)	51 (38–70)

### Cerebrospinal fluid concentration of GFAP and UCHL1 in patients and controls

**Fig. 1:**
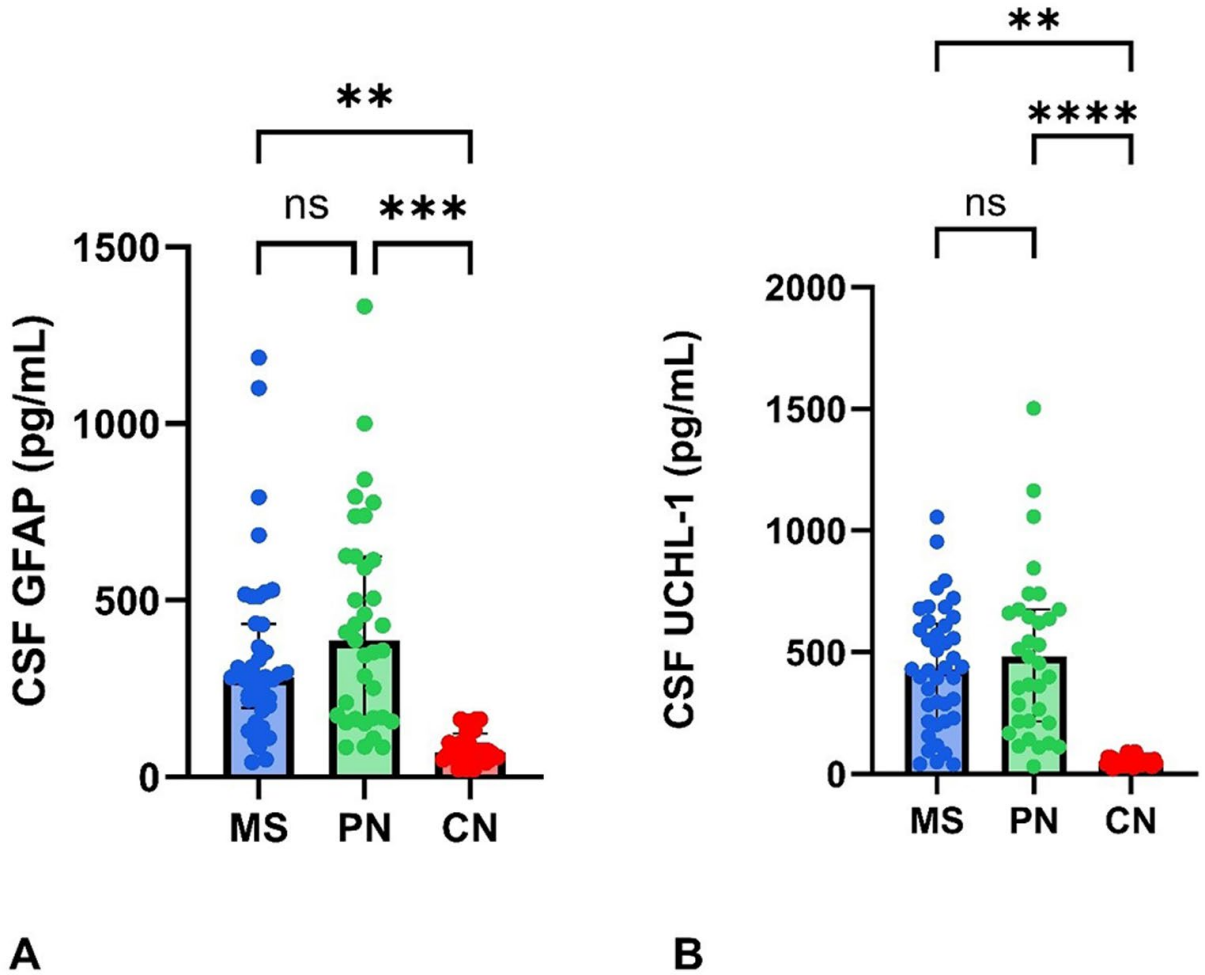
CSF concentration of GFAP (**A**) and UCHL1 (**B**) in patients (MS, *n* = 41; PN, *n* = 35) and controls (*n* = 36). Boxplots showing GFAP and UCHL1 concentrations divided into different neurological diseases displayed on a log_10_-scale y-axis. MS: multiple sclerosis; PN: peripheral neuropathy; CN: control; GFAP: glial fibrillary acid protein; UCHL-1: ubiquitin C-terminal hydrolase; CSF: cerebospinal fluid. ****=*p* < 0.001, *** = *p* = 0.001, **=*p* < 0.01, NS = non-significant

Compared to the control group, median CSF GFAP and UCHL1 concentrations were significantly higher among all patients groups compared to controls, *p* < 0.001, respectively, Table 1., Fig. 1A, B. There was no difference in the CSF concentrations of either molecule between the MS and PN patients.

### Cerebrospinal fluid concentration of GFAP and UCHL1 in MS patients

**Fig. 2:**
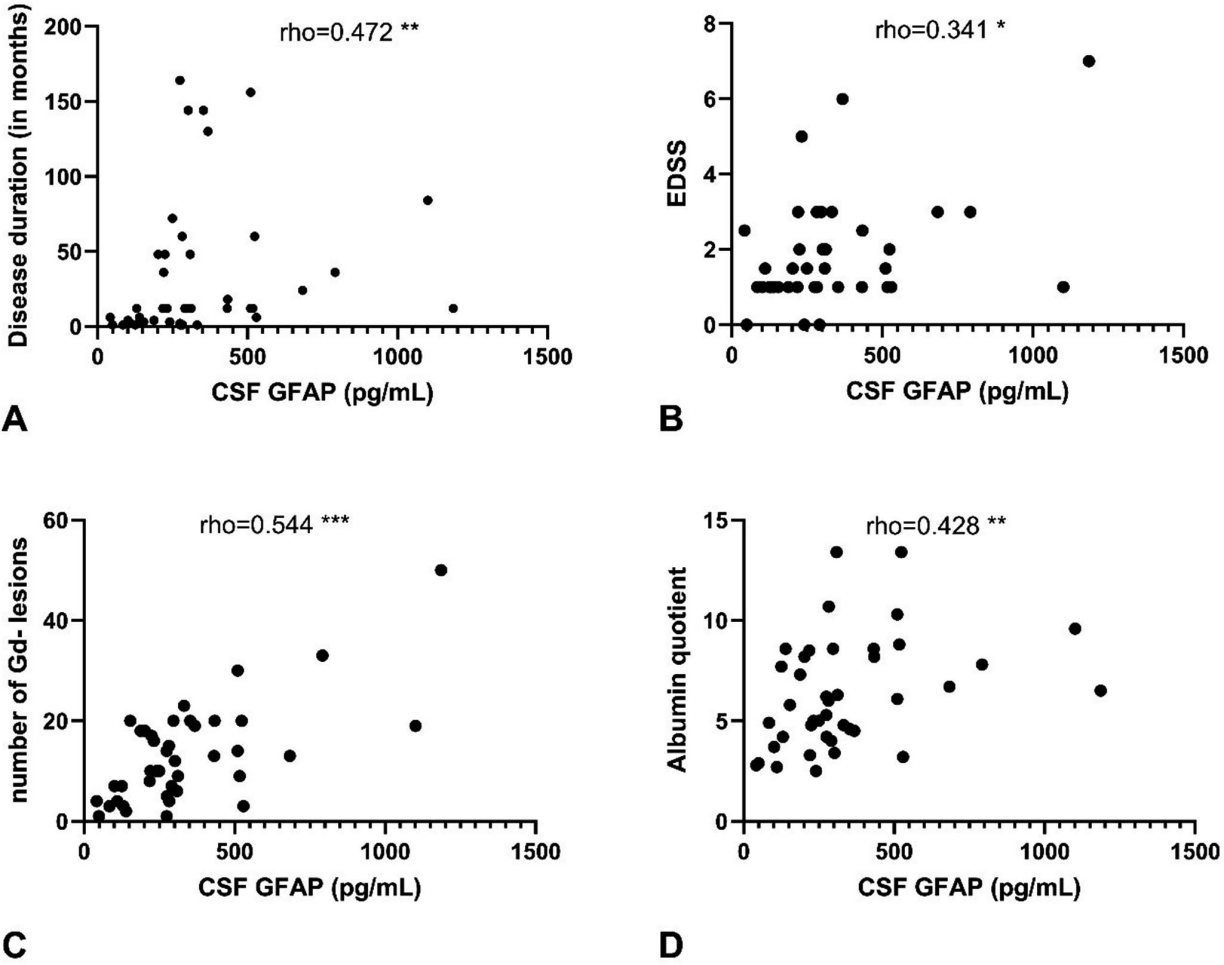
Scatter plots of cerebrospinal fluid GFAP’s Spearman’s rank correlation with clinical markers. CSF GFAP correlation with (**A**) disease duration, (**B**) EDSS, (**C**) number of non-enhancing leasions in MRI of the neuroaxis, (**D**) CSF/blood albumin quotient. GFAP: glial fibrillary acid protein; UCHL-1: ubiquitin C-terminal hydrolase; CSF: cerebospinal fluid; Gd: gadolínium; EDSS: Expanded Disability Status Scale, *** = *p* = 0.001, **=*p* < 0.01, *=*p* < 0.05

**Fig. 3:**
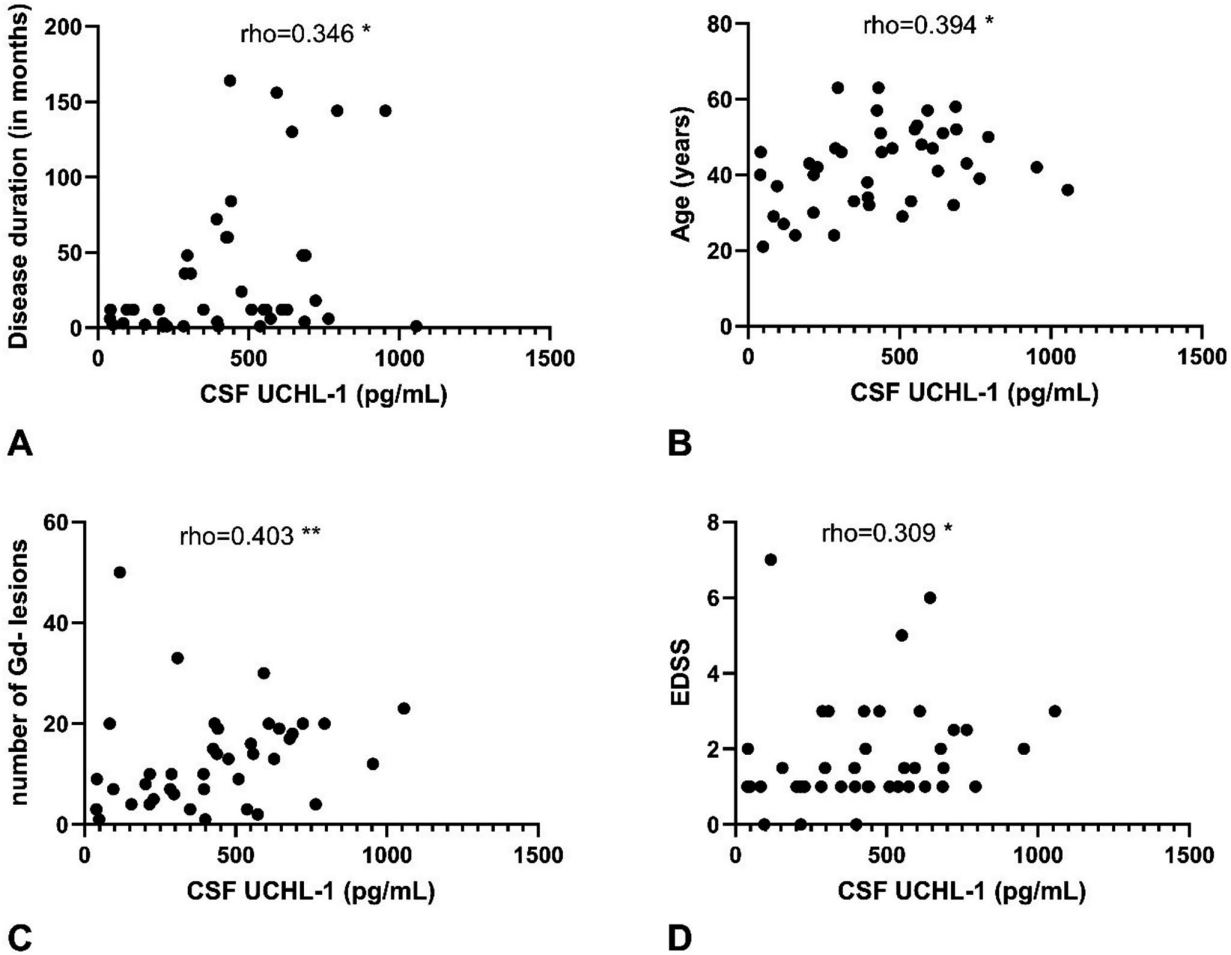
Correlation of cerebrospinal fluid UCHL-1 concentration with (**A**) the number of months from first symptoms until the CSF sample is collected, (**B**) age, (**C**) number of non-enhancing lesions in MRI of the neuroaxis, (**D**) EDSS. GFAP: glial fibrillary acid protein; UCHL-1: ubiquitin C-terminal hydrolase; CSF: cerebospinal fluid; Gd: gadolínium; EDSS: Expanded Disability Status Scale, **=*p* < 0.01, *=*p* < 0.05

**Fig. 4:**
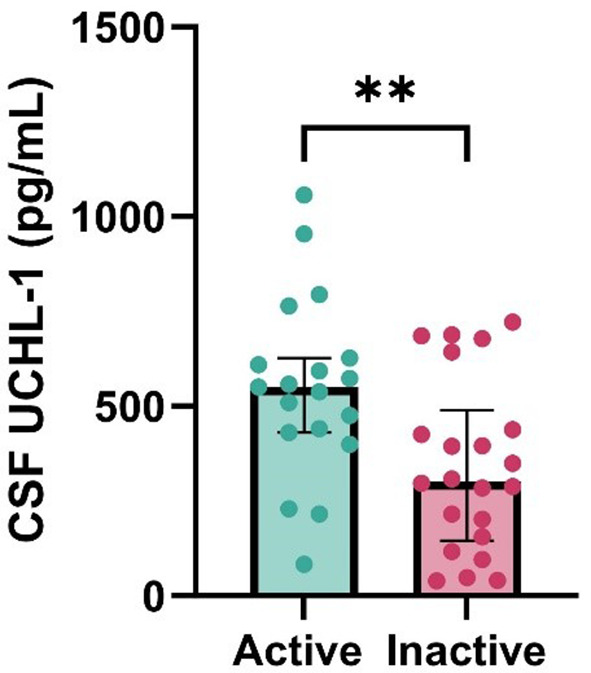
Bar chart showing cerebrospinal fluid UCHL-1 correlation with disease activity. N_active_=17, N_inactive_=24. **=*p* < 0.01, UCHL-1, ubiquitin C-terminal hydrolase. CSF, cerebospinal fluid

In the MS patient group, cerebrospinal fluid GFAP showed the strongest correlation with the number of non-enhancing MRI lesions (Spearman’s rho: 0.544, Fig. 2C) and the duration of disease (Spearman’s rho: 0.472, Fig. 2A). We also observed a significant positive correlation between CSF GFAP and the CSF/serum albumin quotient, as well as between GFAP and the EDSS score (Fig. 2D and B). For CSF UCHL-1, the strongest correlation was observed with the number of non-enhancing MRI lesions (Spearman’s rho: 0.403, Fig. 3C), and less strong positive correlations with the duration of disease and the EDSS compared to GFAP (Fig. 3A and D). For UCHL-1, we observed a significantly higher CSF concentration in patients with enhancing MRI lesions (active disease) compared to those without enhancing lesions (*p* = 0.0097, Fig. 4).

To further analyze the predictive power of GFAP, UCHL-1, and the combination of the two molecules, we converted the number of gadolinium-negative lesions, the EDSS score, and the duration of the disease into binary independent variables based on their median values (0: ≤median, 1: > median).

**Fig. 5:**
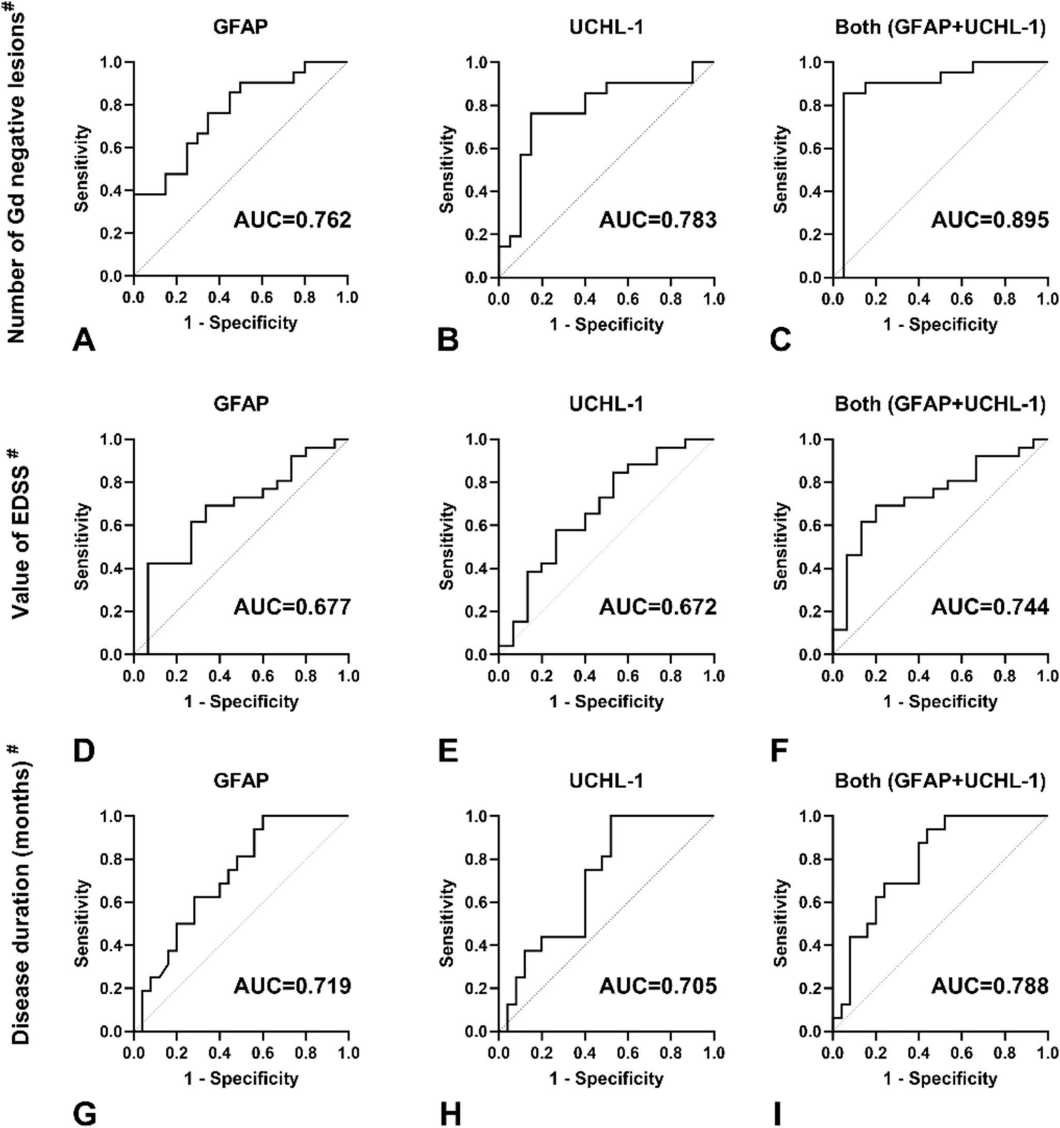
ROC curves for GFAP, UCHL-1 and both in serum to differentiate patients based on the median number of Gd negative lesions (**A, B, C**), value of EDSS (**D, E, F**) and disease duration (**G, H, I**). GFAP, glial fibrillary acid protein, UCHL-1, ubiquitin C-terminal hydrolase, AUC, area under the curve, CI, confidence interval, Gd, gadolínium. Median number of Gd negative lesions: 12, median EDSS: 1.5, median value of disease duration in month: 12

The area under the curve of GFAP and UCHL-1 to differentiate between the median above (12< ) and below (12> ) numbers of Gd negative lesions on MRI were 0.762 (95% CI 0.617 to 0.907) and 0.783 (95% CI 0.634 to 0.983), respectively, Fig. 5. The combination of UCHL1 and GFAP improved diagnostic accuracy (AUC 0.895, 95%CI 0.780 to 1.000) compared with GFAP or UCHL-1 alone, Fig. 5. The AUC values of GFAP, UCHL-1, and their combination in terms of EDSS (D, E,F) and disease duration (G, H, I) are shown in Fig. 5.

### Cerebrospinal concentration of GFAP and UCHL1 in patients with peripheral neuropathy

**Fig. 6:**
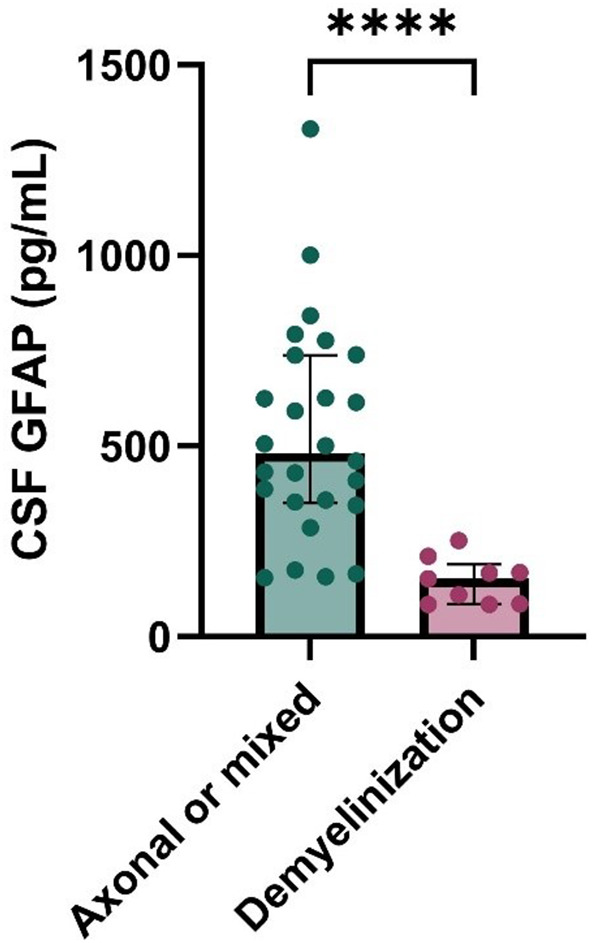
Bar chart showing CSF GFAP levels in patients with axonal or mixed (axonal + demyelinization) peripheral neuropathy and with pure demyelinization. ****=*p* < 0.001. GFAP, glial fibrillary acid protein; CSF, cerebospinal fluid

In neuropathies showing a mixed electrophysiological (axonal + demyelinating) character, the duration of the disease is significantly longer (17 ± 22 months vs. 4 ± 5, *p* = 0.014) compared to neuropathies with purely axonal or demyelinating characteristics. In the group with axonal damage (*n* = 26), motor symptoms predominantly developed slowly, whereas in neuropathies with purely demyelinating characteristics (*n* = 9), rapidly progressive motor symptoms dominated (slowly progressive, 81% vs. rapid progression, 67%, *p* < 0.001). In the patient group with purely demyelinating neuropathy without an axonal component (*n* = 9), the cerebrospinal fluid GFAP level was significantly lower compared to the group with axonal or mixed neuropathy (*n* = 26) (151 pg/L [86–211] vs. 446 [344–625], *p* = 0.001, Fig. 6). Serum GFAP showed a strong positive correlation with both age (rho = 0.465) and the duration of the disease (rho = 0.354). In the case of UCHL-1, we did not observe any meaningful correlation with variables gathered in patients with PN; therefore, the combination of the two markers did not demonstrate statistically significant association with any of the examined parameters.

## Discussion

In our study, we found the following key results:

(i) The levels of GFAP and UCHL-1 in the cerebrospinal fluid (CSF) of MS and PN patients were significantly higher compared to the control group values.
(ii) GFAP levels in the CSF showed a strong correlation with disease severity (EDSS score), the number of non-enhancing MRI lesions, and the duration of the disease in MS patients.
(iii) UCHL-1 levels in the CSF positively correlated with multiple sclerosis disease activity (presence of Gd-enhancing lesions).
(iv) The combined predictive power of these two markers is high for indicating the quantity of non-enhancing MRI lesions, EDSS score and disease duration.
(v) GFAP in the CSF is a sensitive marker of axonal damage in patients with peripheral neuropathy.

Glial fibrillary acidic protein (GFAP), which is expressed by mature astrocytes, has been found to be elevated in the plaques of MS patients, indicating astrocyte damage [33, 34]. A study by Rosengren et al. showed that the CSF assays revealed significantly higher GFAP concentrations in MS patients compared to controls (*p* < 0.01). Additionally, GFAP levels showed a significant correlation with the deficit score (*p* < 0.01), but no correlation was found with exacerbation frequency [35]. Elevated CSF GFAP was associated with long-term MS disease progression according to a recent study [36] while Abdelhak et al. found a positive correlation between CSF GFAP with disease duration in patients with primary progressive MS [37], although GFAP level in serum reflected the disease activity better than CSF GFAP.

According to a recent study, GFAP indicates disease activity in RRMS patients, and its serum levels may correlate with periods of remission or exacerbation. However, its potential to be a biomarker of disease activity is weak [38]. Serum levels of GFAP correlate with the clinical severity scores and MRI lesion count [39]. GFAP serum levels are associated with disease progression in MS [40], correlating with white matter lesion load and inversely correlating with both white matter and grey matter volume [41]. In our study CSF GFAP showed a strong correlation with disease severity, duration of disease and number on non-enhancing lesions on MRI.

Based on the above and the results of our study, the role of GFAP in the management of MS remains unclear. Its role in assessing disease activity and progression is contradictory, and the differing correlations of serum and CSF GFAP with disease markers also urge caution regarding the interpretation of GFAP’s role. A possible explanation for the differing associations between serum and CSF GFAP and disease markers (severity, progression, lesion count, etc.) could be the different sampling times of serum and CSF, the use of different laboratory techniques (SIMOA, ELISA, etc.) for the measurements, as well as the varying transport mechanisms of GFAP [42].

UCHL-1 levels in CSF were significantly higher in patients with Gd-enhancing lesions, i.e., those with active status, compared to patients with only non-enhancing lesions (inactive). However, only GFAP showed a strong correlation with the albumin quotient, which is considered an indicator of BBB damage, whereas UCHL-1 did not. UCHL-1, on the other hand, proved to be sensitive to the appearance of enhancing lesions but did not correlate with their number. GFAP did not show a similar difference between active and inactive patients, but the combined predictive power of the two markers is very high in indicating the quantity of non-enhancing lesions.

Dobson and colleagues observed low UCHL-1 levels in the CSF of patients with multiple sclerosis, which they explained by the sampling occurring in the early stage of the disease [43].

In ALS, serum UCHL-1 levels show a good correlation with CSF UCHL-1 levels, and in addition, serum UCHL-1 levels also show a strong correlation with the duration of the disease [44]. Based on the studies by Sjölin et al. [15], serum UCHL-1 is a good indicator of the location and extent of central nervous system damage, and the concentration of UCHL1 was higher in the cortex than in white matter. The assessment of plasma UCHL1 concentration demonstrated the highest sensitivity for diagnosis (100%) and negative predictive value (100%) in distinguishing between MS patients and healthy individuals [16]. In that study, the plasma UCHL1 concentration was independent of the time of MS relapse and the severity of neurological symptoms. Focal blood-brain barrier (BBB) disruption is detected through gadolinium-enhanced MRI and manifests early in the development of new lesions in multiple sclerosis [45]. The data from van Horssen and colleagues suggest that BBB damage is an early and significant factor in white matter lesions in multiple sclerosis (MS), whereas it is not a critical event in the development of gray matter lesions [46].

However, there is a discrepancy in the literature regarding the extent to which serum and CSF UCHL-1 levels correlate with each other. Alvarez et al. measured UCHL-1 levels in serum and CSF samples from patients with multiple sclerosis using Single Molecule Array (SIMOA) technology and observed a good correlation between UCHL-1 concentrations in the two types of samples [47]. Koerbel et al. did not propose using UCHL-1 serum measurements for monitoring or diagnosing MS, as they found no significant correlations between their serum and CSF levels. This lack of correlation was attributed to the molecular size and structure of UCHL-1, as well as the permeability of the blood-brain barrier in MS patients [48].

However, it is important to note that central nervous system damage can occur independently of BBB disruption and can also lead to significant disability [49]. Gorska et al. [16] indicated that plasma UCHL1 concentration is independent of the time of MS relapse and independent of the severity of neurological symptoms. The discrepancy between the results observed in the previous study (Gorska et al.) and our study regarding UCHL-1 levels and the duration or severity of the disease can be explained by the fact that structural damage caused by grey and white matter lesions may differently affect BBB integrity. This, in turn, can alter the UCHL-1 values detected in the serum. Additionally, it is challenging to quantify the extent to which the observed damage in studies is attributable to grey matter versus white matter damage.The amounts of GFAP and UCHL-1 detectable in serum provide insights into different aspects of the development and progression of MS. Therefore, their combined examination and interpretation could offer practical clinical benefits.

Overall, the combined predictive power of the two molecules surpasses their individual predictive power in indicating the number of gadolinium-negative lesions, the EDSS score, and the duration of the disease. To our knowledge, this result has not yet been published, making it potentially significant for more accurately predicting the course of MS and selecting drug treatments, provided our findings can be reproduced in a larger cohort.

Considering the conflicting results available in the literature, the use of GFAP and UCHL-1 in clinical practice for MS patients is not anticipated at present, given the uncertain correlation between serum and CSF values, as well as the non-standardized sampling times and techniques.

In our study, GFAP levels in CSF showed a strong correlation with the presence of electrophysiologically confirmed axonal damage in patients with peripheral neuropathy. Our results are consistent with previous findings reported in the literature. In patients with severe axonal degeneration have higher GFAP expression than patients with demyelinating neuropathies according to studies from early 90s [50, 51]. Serum GFAP levels were increased in axonal GBS compared with controls and AIDP and were correlated with 6 month functional outcome [52]. During Wallerian degeneration, Schwann cells (SCs) lose their connection with axons, undergo dedifferentiation, and re-enter the cell proliferation cycle. They begin to express surface molecules typical of embryonic development and show increased levels of cytoskeletal proteins like GFAP. GFAP released by Schwann cells enters the adjacent fluid compartment and can be detected in the serum [21]. This could be the potential mechanism for the increase in serum GFAP levels associated with axonal components.

Unfortunately, in our study, CSF UCHL-1 did not show significant correlation with the clinical and electrophysiological data of patients with peripheral neuropathy. Therefore, we were unable to assess the combined predictive power of the two molecules. However, based on previous literature [19, 53], UCHL-1 may play a role in the diagnosis and prognosis of peripheral neuropathies.

A previous study with TBI patients [54] revealed a strong correlation between CSF and serum UCHL-1, providing additional support for the possibility of using only serum samples to analyze this biomarker. A similar correlation is observed between serum and CSF levels of GFAP in patients with amyloid angiopathy [55]. Moreover, serum GFAP had a higher discrimination of Aβ burden than CSF GFAP, irrespective of freeze-thaw cycles [56], suggesting that GFAP release into the bloodstream can be just as effective an indicator of central nervous system pathological processes as the much more cumbersome and invasive CSF measurement.

Our study has some limitations. The number of study subjects is relatively low, and no follow-up was conducted after the baseline examination. In this study, the relatively low number of items analyzed limits the statistical power, potentially reducing the likelihood of detecting true effects or associations. Consequently, results may be subject to a higher risk of Type II errors (false negatives), where meaningful associations remain undetected. Furthermore, confidence intervals around estimated effects may be wider, reflecting greater uncertainty in the findings. The results should be interpreted with caution, and further studies with larger sample sizes are recommended to confirm our findings. In the group of patients with multiple sclerosis, since the sampling was done as part of the diagnostic evaluation, it was not possible to assign them to a subgroup. We did not measure serum GFAP and UCHL-1 levels in our cohort, so we could not compare them with the values measured in the CSF. The role of GFAP and UCHL-1, as they are non-specific markers, would primarily be in prognosis; however, given that the sampling and diagnosis occurred at nearly the same time, we did not examine this in our current study. In summary, the combined testing of GFAP and UCHL-1, as it highlights different pathological processes of MS, could be useful in clinical practice, although further prospective studies are needed in this area. Based on our study, there is no advantage of combined testing of the two molecules compared to testing GFAP alone in patients with peripheral neuropathies.

## Data Availability

Data supporting the fndings of this study are available upon reasonable request.
